# Microbial Light-Activatable Proton Pumps as Neuronal Inhibitors to Functionally Dissect Neuronal Networks in *C. elegans*

**DOI:** 10.1371/journal.pone.0040937

**Published:** 2012-07-16

**Authors:** Steven J. Husson, Jana F. Liewald, Christian Schultheis, Jeffrey N. Stirman, Hang Lu, Alexander Gottschalk

**Affiliations:** 1 Buchmann Institute for Molecular Life Sciences, Johann Wolfgang Goethe-University Frankfurt, Frankfurt am Main, Germany; 2 Institute of Biochemistry, Johann Wolfgang Goethe-University Frankfurt, Frankfurt am Main, Germany; 3 Interdisciplinary Bioengineering Program, School of Chemical & Biomolecular Engineering, Georgia Institute of Technology, Atlanta, Georgia, United States of America; Harvard University, United States of America

## Abstract

Essentially any behavior in simple and complex animals depends on neuronal network function. Currently, the best-defined system to study neuronal circuits is the nematode *Caenorhabditis elegans*, as the connectivity of its 302 neurons is exactly known. Individual neurons can be activated by photostimulation of Channelrhodopsin-2 (ChR2) using blue light, allowing to directly probe the importance of a particular neuron for the respective behavioral output of the network under study. In analogy, other excitable cells can be inhibited by expressing Halorhodopsin from *Natronomonas pharaonis* (NpHR) and subsequent illumination with yellow light. However, inhibiting *C. elegans* neurons using NpHR is difficult. Recently, proton pumps from various sources were established as valuable alternative hyperpolarizers. Here we show that archaerhodopsin-3 (Arch) from *Halorubrum sodomense* and a proton pump from the fungus *Leptosphaeria maculans* (Mac) can be utilized to effectively inhibit excitable cells in *C. elegans*. Arch is the most powerful hyperpolarizer when illuminated with yellow or green light while the action spectrum of Mac is more blue-shifted, as analyzed by light-evoked behaviors and electrophysiology. This allows these tools to be combined in various ways with ChR2 to analyze different subsets of neurons within a circuit. We exemplify this by means of the polymodal aversive sensory ASH neurons, and the downstream command interneurons to which ASH neurons signal to trigger a reversal followed by a directional turn. Photostimulating ASH and subsequently inhibiting command interneurons using two-color illumination of different body segments, allows investigating temporal aspects of signaling downstream of ASH.

## Introduction

Optogenetic technologies use light to gain exogenous control (e.g. activation by the depolarizing ChR2 and inhibition by the hyperpolarizing NpHR) of defined cells or tissues in a non-invasive manner [1]–[7]. Recently, an extensive screen of type I microbial opsins from Archaebacteria, Bacteria, plants and fungi revealed outward-directed proton pumps as useful alternatives to achieve neuronal inhibition [8]. The yellow-green light-sensitive archaerhodopsin-3 (Arch) [9] appears to be more powerful than NpHR. Another proton pump, Mac [10], enables neuronal silencing by green-blue light [8]. This opens the possibility to inhibit different neuronal populations, depending on the illumination wavelengths used.

The nematode *C. elegans* is highly amenable to study fundamental principles of network function by optogenetics due to its transparency, ease of genetic manipulation and its well-defined nervous system. *C. elegans* contains exactly 302 neurons which are connected by synapses and/or gap junctions that have been mapped by serial reconstruction of electron micrographs [11]. Despite these advantages, it remains a challenging task to characterize small neuronal networks by optogenetics in the nematode. Several recent reports describe the use of ChR2 to photoactivate neurons in *C. elegans* [3], [5], [6], [12]–[14]. Although NpHR has been used in the nematode to hyperpolarize muscle cells or neurons [7], [13], [15], [16], it is not such a robust tool, as the protein traffics inefficiently to the plasma membrane in eukaryotic cells. This problem was overcome in mammalian tissues by adding ER export and trafficking signals [17], [18]. However, as these signals are not conserved, they did not improve trafficking in *C. elegans* (**Figure S1**). Achieving neuronal inhibition by using NpHR in *C. elegans* is thus only possible when strong promoters for the cells of interest are available, which hampers network analysis in this important model system. Consequently, no “circuit-breaking” experiments have been described, i.e. to simultaneously photoactivate neurons by ChR2 and to inhibit downstream neurons using NpHR. This would be a very powerful strategy to study the contribution of each individual neuron in the circuit of interest, and would allow characterizing which downstream neurons are required for processing and integration of perceived signals.

Until recently, it was also difficult to apply different colors of light to defined body regions in a freely behaving animal, rendering the simultaneous use of ChR2 and NpHR experimentally challenging. To overcome these difficulties, we previously developed a multimodal illumination technology to simultaneously project light of various colors on different defined body segments of a freely moving worm [6], [19]. In addition, this setup allows simultaneous tracking and recording of the evoked behavior, enabling us to further probe the possibilities of other optogenetic reagents.

Here, we first explored and characterized the use of Mac and Arch as alternatives to NpHR for hyperpolarization of *C. elegans* body wall muscle cells (BWMs), cholinergic neurons and command interneurons, downstream of photoactivated mechanoreceptor neurons. Next, we analyzed a small nociceptive network including the polymodal ASH neurons that detect diverse chemical repellents and mechanical touch to the nose [20]–[23]. ASH mainly signals through the backward command interneurons AVA, AVD and AVE [11] to evoke a reversal and subsequently a directional (“omega”) turn. We photoactivated ASH *via* ChR2, “titrating” the light stimulus to induce the entire behavioral program, or only its initiation (i.e. reverse locomotion), and used Mac or Arch to affect downstream activity in the command interneuron circuit. Temporal parameters of the behavioral output depended on the illumination protocol used.

## Results

### Efficiency of Modified NpHR-variants for Optogenetic Hyperpolarization of Muscle Cells

As the use of NpHR is not efficient to achieve neuronal inhibition in *C. elegans*, we introduced a variety of ER export and trafficking signals in an attempt to improve expression, similar to optimized variants for mammalian tissues [17], [18]. Different NpHR variants were expressed in BWMs of *C. elegans* and resulting relaxation effects upon yellow light exposure were measured and compared with the wild type NpHR (WT-NpHR or later referred to as NpHR; **Figure S1**).

An alternative start codon 57 bp upstream of the NpHR start codon in the genome of *Natronomonas pharaonis*, results in the addition of 19 amino acids (termed *native signal sequence*) that can be recognized as putative eukaryotic signal sequence. We tested whether this sequence would enhance cell surface expression of NpHR. However, we observed even reduced relaxation effects when comparing this construct with WT-NpHR. In 2008, Gradinaru and colleagues described a human codon optimized NpHR variant with an additional signal sequence from a nicotinic acetylcholine receptor (nAChR) subunit at the N-terminus and an endoplasmic reticulum (ER) export motif from the Kir2.1 inward rectifying potassium channel that displayed enhanced cell surface expression (eNpHR2.0) [17]. However, compared to WT-NpHR, expression and activation of eNpHR2.0 resulted in smaller relaxation effects, as if addition of mammalian export motifs even reduced the hyperpolarization efficiency (**Figure S1B**).

We therefore looked for *C. elegans* motifs putatively promoting ER exit. The rat dopaminergic D1 receptor contains a motif following transmembrane helix 7 (TM7) that was recently shown to be crucial to promote transport of the receptor to the cell surface [24]. As this motif is conserved in the *C. elegans* D1-type DOP-1 receptor, we generated NpHR constructs including this motif either after TM7 or fused to the C-terminus of the WT-NpHR::eCFP construct. In this context, we optionally also included the native signal sequence at the N-terminus. However, only when the motif was added to the C-terminus and the intrinsic signal sequence was included, relaxation effects were similar to WT-NpHR, while the other NpHR variants were less efficient (**Figure S1B**). As hyperpolarization might become saturating at high light intensities, we compared both constructs at different light intensities from 20 µW/mm^2^ to 10 mW/mm^2^. Evoked relaxation effects of both variants were basically indistinguishable for all intensities tested, indicating that utility of intrinsic signal and *C. elegans*-specific trafficking sequences did not enhance NpHR’s ability for optogenetic hyperpolarization of excitable cells in *C. elegans*.

### Photoinhibition of *C. elegans* Muscles by Arch or Mac

**Figure 1:**
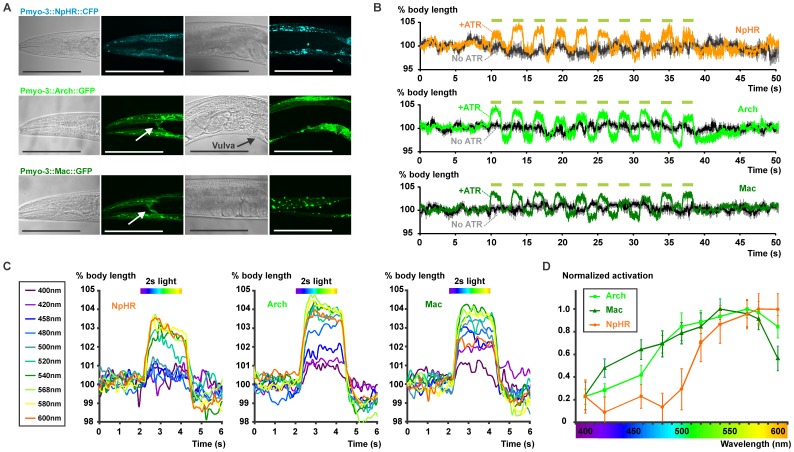
Expression and photoactivation of NpHR, Arch and Mac in body wall muscle cells. **A,** NpHR::CFP (upper panels), Arch::GFP (middle panels) and Mac::GFP (lower panels) were expressed in body wall muscle cells (BWMs) of *C. elegans* using the promoter of the *myo-3* gene, as shown by confocal imaging. Scale bars represent 100 µm; white arrows point to muscle arms. **B**, Relative body lengths of animals expressing either NpHR (upper panel, n = 10), Arch (middle panel, n = 15) or Mac (lower panel, n = 18) were monitored when challenged with 10 yellow-green (0.96 mW/mm^2^, 540–580 nm, **Figure S2**) light pulses of 1.5 s with a 1.5 s ISI. **C,** Body elongations of animals expressing NpHR (left panel), Arch (middle panel) or Mac (right panel) were measured upon illumination with a 2 s light pulse (0.14 mW/mm^2^) with different wavelengths, as indicated in the legend (**Figure S3**). **D**, Activity spectra of the three hyperpolarizers were constructed by plotting the normalized (maximal elongation is set to 1 for each strain) average body elongations for each wavelength, calculated for a 1.5 s timeframe, starting 0.5 s after the onset of the light until the end of the light pulse. Error bars display SEM values.

To investigate whether Arch and Mac [8] can be used as hyperpolarizers in *C. elegans*, as alternatives to the well-established NpHR [7], we first expressed NpHR::CFP, Arch::GFP and Mac::GFP in BWMs using the muscle-specific *myo-3* promoter. Fluorescence could easily be observed in the muscle cells and membranous muscle arm extensions by confocal microscopy (**Figure 1A**). Animals expressing very high levels of Mac or Arch (80 ng/µL injected DNA) exhibited slightly sluggish locomotion. To avoid this, we aimed for lower expression levels (10 ng/µL injected DNA), which upon illumination with yellow-green light (0.96 mW/mm^2^, 540–580 nm, **Figure S2**) resulted in a robust elongation of the animals as BWMs relaxed. We challenged animals expressing NpHR (100 ng/µL injected DNA), Arch or Mac (both 10 ng/µL injected DNA) in BWMs with 10 successive pulses of 1.5 s yellow-green light with an interstimulus interval (ISI) of 1.5 s and calculated the percentage of length increase by automated video analysis [3]. This revealed reproducible elongations to about 104% of the body length for animals from all three strains (**Figure 1B** **, Video S1**). As nematodes do not contain sufficient levels of the rhodopsin co-factor all-*trans* retinal (ATR), we supplemented the bacterial food with ATR, as established for ChR2 and NpHR [5], [7]. When ATR was omitted from the growth media, no length changes were observed (gray traces in **Figure 1B**).

In order to compare the sensitivity to light of different wavelengths, we measured body elongations of worms expressing NpHR, Arch or Mac evoked by 2 s of light (0.14 mW/mm^2^) for 10 different wavelengths between 400 and 600 nm, using excitation band-pass filters (**Figure 1C** **, Figure S3**). We compared the maximal achieved elongations for each hyperpolarizer during the illumination (NpHR: 3.0±0.4% at 580 nm; Mac: 3.9±0.4% at 540 nm and Arch: 4.2±0.3% at 568 nm; significantly different compared to NpHR with *p<0.05). Animals expressing NpHR showed the smallest effect, indicating that Mac and Arch either were more efficiently inserted into the plasma membrane, or were more light sensitive, or had higher pump currents than NpHR. Our findings in *C. elegans* are thus in line with previous recordings showing that Arch-mediated photocurrents were larger than those evoked by NpHR [8]. Thus Mac and Arch, depending on the particular application, appear to be the hyperpolarizing reagents of choice.

We determined the action spectra of NpHR, Arch and Mac, in the *C. elegans* background, by plotting the normalized average body elongations over the whole range of wavelengths (**Figure 1D**). As in other model systems, we observed maximum NpHR activity using 580 and 600 nm light (no longer wavelengths were tested here), whereas animals expressing Arch showed maximum elongations at 568 nm. The peak of the Mac action spectrum was observed at 540 nm, which is more blue-shifted compared to Arch and NpHR. The spectrum of Mac also had a more pronounced shoulder in the blue region. As a consequence, animals expressing Mac responded to a wider range of wavelengths; from yellow to blue light. Therefore, Mac-triggered neuronal inhibition can simultaneously be achieved with ChR2-evoked depolarization (e.g. in other neuronal populations), using the same color of light (blue). Indeed, 64±9% of the peak hyperpolarizing power of Mac can be evoked using a 458 nm band-pass filter, whereas Arch yields 42±9% and NpHR only shows 23±10% of its maximal activity at this wavelength.

**Figure 2:**
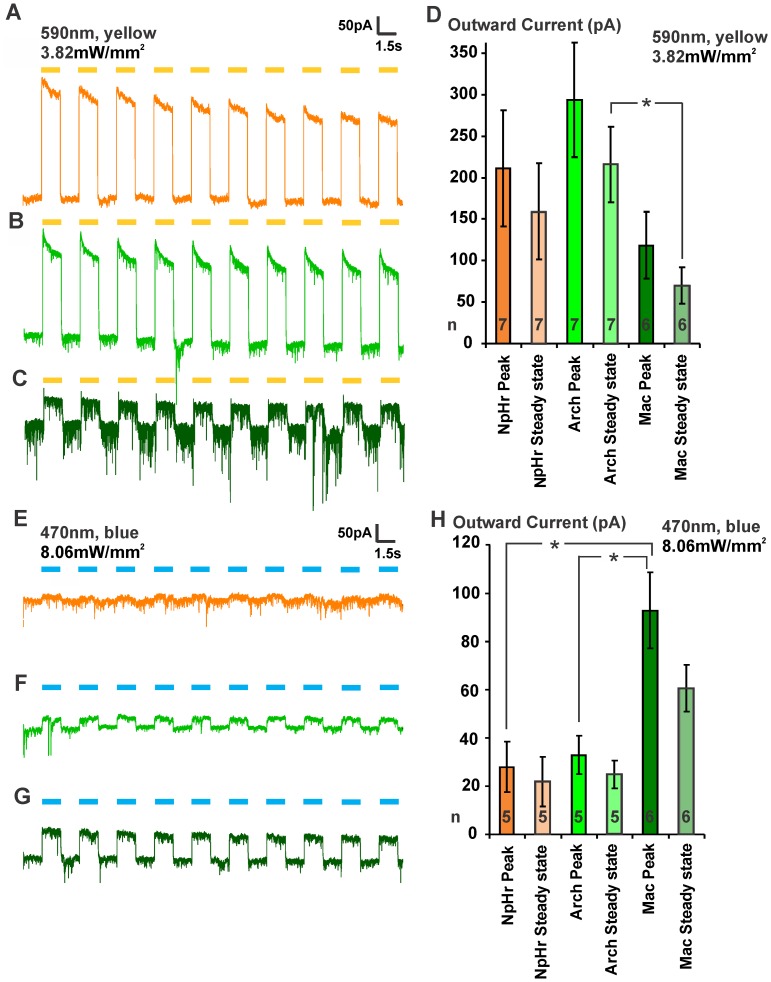
Comparison of outward currents evoked by NpHR, Arch and Mac. **A–D,** 10 yellow (3.82 mW/mm^2^, 590 nm, **Figure S4**) light pulses of 1.5 s were applied with a 1.5 s interstimulus interval on dissected worms expressing either **A**, NpHR, **B**, Arch or **C**, Mac in BWMs. The evoked outward currents were measured by patch clamping muscle cells. Representative traces are shown in **A**–**C**. **D**, Mean outward current values for the peak values and steady state are displayed for the first light pulse. Mean input resistances (GOhm) were as follows: NpHR before light: 0.68±0.22; NpHR during light: 0.23±0.08; Arch before light: 0.49±0.15; Arch during light: 0.12±0.03; Mac before light: 0.91±0.20; Mac during light: 0.38±0.12. **E–H,** In analogy, 10 blue (8.06 mW/mm^2^, 470 nm, **Figure S4**) light pulses were applied and evoked currents were monitored by electrophysiology. Mean input resistances (GOhm): NpHR before light: 1.05±0.53; NpHR during light: 0.40±0.13; Arch before light: 1.48±0.39; Arch during light: 0.67±0.14; Mac before light: 1.02±0.33; Mac during light: 0.33±0.09. Error bars and ± indicate SEM values; statistical significance was determined using student’s t test; *p<0.05.

Next, we used muscle whole-cell voltage clamp recordings on dissected animals as a more direct approach to monitor photocurrents (**Figure 2**). To compare the maximal outward currents for the three hyperpolarizers, we used saturating light intensities. We applied 10 successive pulses of 1.5 s yellow light (3.82 mW/mm^2^, 590 nm, **Figure S4**) with an ISI of 1.5 s (**Figure 2A–C**). Although the maxima for Arch and Mac were reported to be more blue-shifted [8], which is in agreement with the action spectra that we compiled, Arch evoked stronger outward currents at this wavelength compared to NpHR and Mac (**Figure 2D**). In contrast, when using blue light (8.06 mW/mm^2^, 470 nm, **Figure S4**), Mac achieved significantly higher outward currents compared to Arch or NpHR (**Figure 2E–H**), in agreement with our behavioral data. Taken together, Arch is a more potent tool for optical hyperpolarization compared to NpHR, whereas its counterpart Mac is the favorable choice when considering concomitant activation of ChR2 with blue light (primary peak at 460 nm in the action spectrum).

### Optical Silencing of Cholinergic Motor Neurons by Arch and Mac

**Figure 3:**
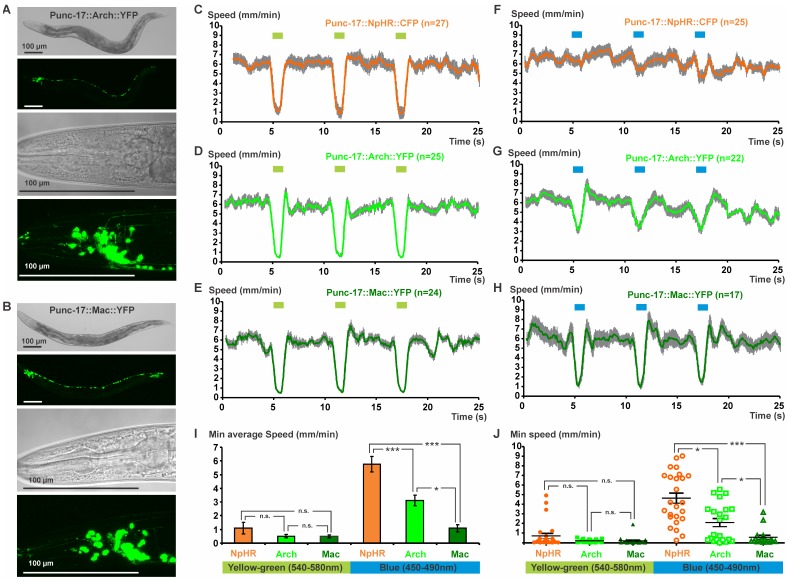
Inhibition of cholinergic neurons by NpHR, Arch and Mac affects locomotion. **A,** Arch::YFP and **B,** Mac::YFP were expressed in cholinergic neurons using the promoter sequence of the *unc-17* gene, as visualized by confocal imaging. Scale bars indicate 100 µm. Animals expressing **C**, NpHR, **D**, Arch or **E**, Mac in the cholinergic neurons were challenged with three yellow-green (0.52 mW/mm^2^, 540–580 nm, **Figure S2**) light pulses of 1 s, with a 5 s ISI. The effect on the locomotion was analyzed by automated video analysis and the resulting speed graphs indicate the mean speed with SEM values. **F–H**, In analogy, the same illumination protocol was performed with blue light (0.41 mW/mm^2^, 450–490 nm, **Figure S2**). **I**, Minimal average speeds and **J**, minimal achieved speeds for the individual worms are indicated for the first light pulse of the two different illumination protocols. Statistical analysis was performed using a non-parametric Kruskal-Wallis test followed by a Dunn’s *post-hoc* test; ***p<0.0001, *p<0.05, n.s. not significant.

We next assessed if Arch and Mac can be used as neuronal silencers in *C. elegans*. Arch and Mac were expressed in cholinergic motor neurons using the *unc-17* promoter, as described previously for NpHR::CFP [7], i.e. in neurons innervating ventral or dorsal muscles, respectively [11] (**Figure 3A,B**). Animals expressing NpHR in cholinergic neurons reduced or stopped swimming behavior upon illumination with yellow light [7]. Here we measured the effect of NpHR-, Arch- or Mac-triggered optical inhibition of cholinergic neurons on the worm’s locomotion speed on agar substrate, using video analysis software [25] that we combined with a shutter control program. Transgenic animals were challenged with three yellow-green light pulses of 1 s (540–580 nm, 0.52 mW/mm^2^, 5 s ISI; **Figure 3C–E** **, Video S2**, **Figure S2**). This illumination protocol evoked an immediate inhibition of forward movement for all three strains, demonstrating their utility as potent neuronal hyperpolarizing reagents, i.e. being capable of silencing neurons effectively (**Figure 3I,J**). When blue light was used (450–490 nm, 0.41 mW/mm^2^; **Figure 3F–H** **, Figure S2**), animals expressing Mac showed the strongest inhibition of locomotion, followed by the worms expressing Arch. In contrast, animals expressing NpHR did not respond to this wavelength (**Figure 3I,J**). These behavioral assays are in agreement with the action spectra we measured and indicate that Mac is suitable for neuronal hyperpolarization with blue light, for example in combination with optical activation of another subset of neurons using ChR2. When inhibition independent of ChR2 activation is required, Arch would be the more suitable reagent. In addition, selective illumination allows activating or inhibiting specific neurons, provided they are located at some distance to each other in the worm’s body.

### Comparing NpHR, Mac and Arch to Interfere with Downstream Signaling from the Touch Receptor Neurons

**Figure 4:**
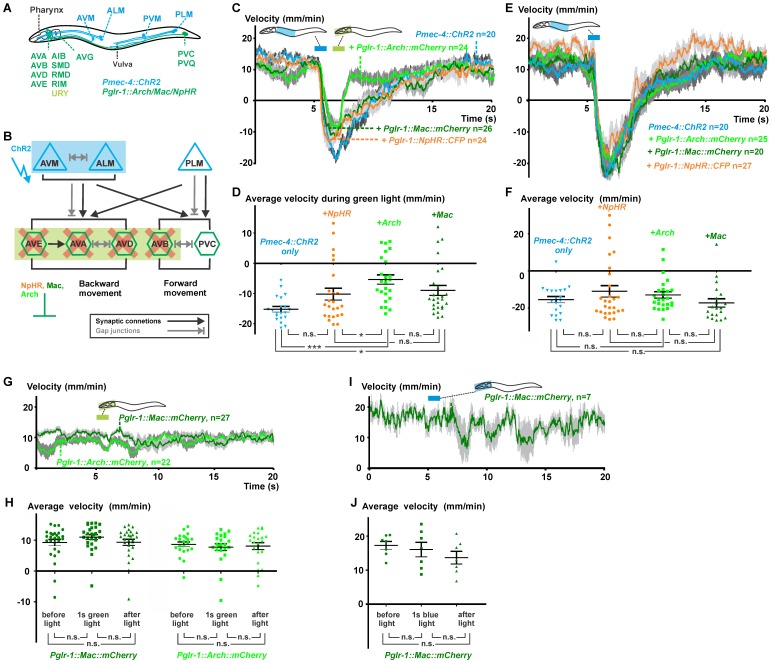
Evaluation of NpHR, Arch and Mac as tools to inhibit downstream signaling in the gentle touch circuit. **A**, Schematic representation of a transgenic animal expressing ChR2 in the touch receptor neurons (*Pmec-4::ChR2)* and either Arch, Mac or NpRH in downstream command interneurons (and other cells, driven by the *glr-1* promoter [28]) that coordinate forward and backward movement. The location of the six touch receptor neurons (blue) and the cells expressing *glr-1* are indicated; URY is displayed in light green as it expresses *glr-1* only weakly. **B**, Simplified wiring diagram of the gentle touch network based on [11], indicating the synaptic connections and gap junctions. The blue and green boxes indicate which neurons were photoactivated (blue) or inhibited (green). **C**, Using selective illumination, the AVM and ALM mechanosensory neurons were photoactivated with blue light (4.2 mW/mm^2^, 435–475 nm, **Figure S5**) for 1s by illumination of the indicated body segment. 0.5 s after the first blue light pulse, we applied a green light pulse (4.7 mW/mm^2^, 543–593 nm, **Figure S5**) of 1s on the head region of the animals; mean velocity with SEMs are indicated for different genotypes. **D**, Average velocities of each worm during the 1 s green light pulse is plotted. Statistical analysis was performed using a non-parametric Kruskal-Wallis test followed by a Dunn’s *post-hoc* test; ***p<0.0001, **p<0.005, *p<0.05, n.s. not significant. **E**, As a control, we only photoactivated AVM and ALM with blue light (as in C, but no green pulse was given) and plotted mean velocities with SEMs for all different genotypes. **F**, Average velocities of each worm for a 3 s time bin (6s–9s) after the blue light pulse is plotted. **G**, Animals expressing Arch or Mac in the command interneurons were challenged with the green light pulse of 1 s on the head; no ChR2 is present in these worms. Average velocity traces and SEM values (gray shade) are shown. **H**, Average velocities of each individual worm for three time-bins are plotted: 5 s before illumination, during the 1 s green light pulse, and 10 s after illumination. Data were further analyzed using a non-parametric repeated measures test (Friedman), followed by a Dunn’s multiple comparison test. **I**, In analogy, head regions of animals expressing Mac in the command cells were illuminated with a 1 s blue light pulse. **J**, Resulting average velocities for the three time bins as indicated in (**H**) are shown.

*C. elegans* contains six touch receptor neurons (TRNs) that respond to gentle mechanical touch (e.g. by an eyebrow hair) to different regions of the body [26], [27] (**Figure 4A**). The TRNs make direct contact *via* synapses and gap junctions with the forward and backward command interneurons that integrate the information from the different TRNs (and other cells) and shape the behavioral output as a forward or backward movement (**Figure 4B**). Using ChR2 expressed in the TRNs, gentle touch responses to various regions of the body can be mimicked by illuminating distinct body segments harboring the respective TRNs [6]. For example, illumination of the second quarter and thereby photoactivating AVM and ALML/R cell bodies and part of their axons caused the animals to reverse. In animals also expressing *Mac::mCherry* from the *glr-1* promoter in the command neurons AVA, AVB, AVD, AVE and PVC (as well as in additional cells; AIB, RMD, RIM, SMD, AVG, PVQ and weakly in URY [28]), this reversal response could be attenuated by illumination of the head using green light, thereby hyperpolarizing the command neurons *via* Mac [6].

Here we compared the three hyperpolarizing alternatives using a similar experimental setup (**Figure 4C**). Animals expressing the genomically integrated transgene *ljIs123* (encoding *Pmec-4::ChR2*) were complemented with additional extrachromosomal transgenes, encoding either *Pglr-1::NpHR::CFP, Pglr-1::Mac::mCherry* or *Pglr-1::Arch::mCherry.* A 1 s blue light pulse (4.2 mW/mm^2^, 435–475 nm, **Figure S5**) photoactivating AVM and ALML/R initiated robust reversals, termination of which and return to forward locomotion could abruptly be induced by the hyperpolarizing action of Arch in the command neurons in the head. We applied the 1 s yellow-green light pulse (4.7 mW/mm^2^, 543–593 nm, **Figure S5**) when the worms reached their maximal negative velocity (fastest backward movement), which was ∼0.5 s after the blue light pulse. Mac and NpHR evoked similar, but clearly less pronounced effects compared to Arch. Worms expressing Mac or NpHR in the downstream neurons in the head reached mean maximal negative velocities of about −12 mm/min, whereas *Pmec-4::ChR2* animals without hyperpolarizers showed mean maximal negative velocities of −18 mm/min. In contrast, only −8 mm/min could be achieved when Arch was present in the command interneurons, indicating an efficient and strong inhibition of the backward movement. In addition, these worms already moved forward again (positive velocity comparable with the initial starting speed) 0.5 s after the yellow-green light pulse. In contrast, Mac or NpHR-containing animals still showed a mean negative velocity at this time point (*Pmec-4::ChR2* −9.8±2.8 mm/min n = 20, *Pmec-4::ChR2; Pglr-1::NpHR::CFP* 9.1±1.9 mm/min n = 24, *Pmec-4::ChR2; Pglr-1::Mac::mCherry* –5.3±2.2 mm/min n = 26, *Pmec-4::ChR2; Pglr-1::Arch::mCherry* 8.4±1.7 mm/min n = 24) (**Figure 4C**). We plotted the average velocities of individual animals during the yellow-green light pulse and observed significant differences between the three hyperpolarizers as tools to inhibit downstream signaling (**Figure 4D**). To rule out that the first blue light pulse is acting on the hyperpolarizers (as some neurites from the *glr-1-*expressing neurons are located in the path of the blue light), we applied the blue light pulse only. In this case, all strains showed similar reversals, emphasizing that no major unwanted co-inhibition of *glr-1* positive cells by blue light occurred (**Figure 4E,F**).These data again indicate the superiority and fast hyperpolarizing action of Arch and its usefulness in inhibiting neurons downstream from ChR2-photoactivated neurons.

We next wanted to investigate if our observations are real circuit breaking effects instead of just cancelling out of two opposing behaviors, e.g. because inhibiting interneurons could per se evoke a behavioral response. We expressed Arch or Mac in the interneurons (no ChR2 is present in these lines) and continuously measured the velocity of the worms before, during and after applying the 1 s yellow-green light pulse on the head region of the animals (**Figure 4G**). No statistically significant differences in average velocity could be observed for the three time bins (**Figure 4H**), as was also the case when challenging animals expressing *Pglr-1::Mac* with a blue light pulse on the head (**Figure 4I,J**). Thus, inhibition of *glr-1*-expressing neurons (including command neurons) in the head does not per se evoke forward locomotion. Overall, our data illustrate that signaling within a neuronal circuit, in which a sensory (or other) neuron is photoactivated *via* ChR2, can be interrupted by inhibition of downstream nodes of the network, using hyperpolarizing optogenetic tools.

### Analyzing the Nociceptive ASH Network by Optogenetics

**Figure 5:**
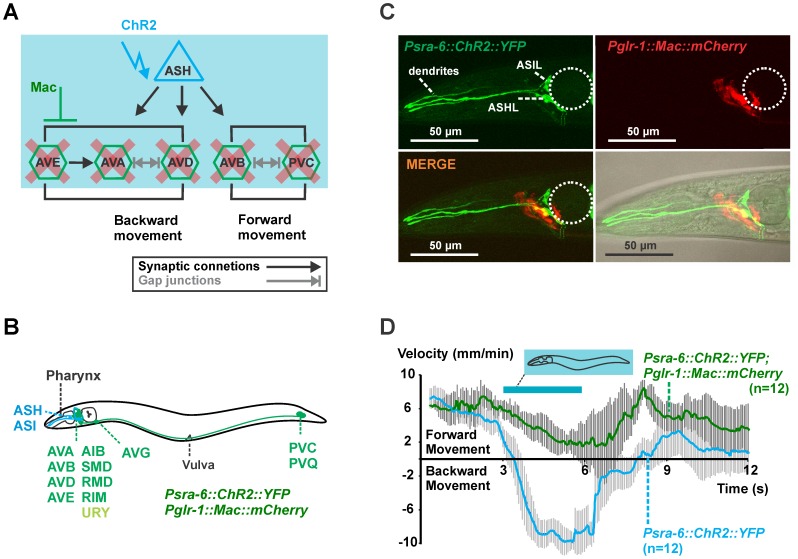
One-color optogenetic analysis of the ASH circuit by ChR2 and Mac. **A,** Simplified representation of the wiring diagram of the polymodal nociceptive neuron ASH with indication of the synaptic contacts and gap junctions according to [11]. **B**, Scheme of a worm expressing ChR2 in ASH and Mac or Arch in downstream cells, driven by the promoter of the *glr-1* gene. **C**, Confocal imaging of the head region of animals expressing *Psra-6::ChR2::YFP* (expression of ChR2 in ASH and ASI, green) and *Pglr-1::Mac::mCherry* (expression of Mac in the command interneurons, red). The location of the terminal bulb of the pharynx is indicated by the dotted circle. **D**, Animals expressing ChR2 in ASH and ASI (*Psra-6::ChR2::YFP*, blue trace) and animals that also expressed Mac in the downstream command interneurons (*Psra-6::ChR2::YFP; Pglr-1::Mac::mCherry*, green trace) were challenged with blue light (0.41 mW/mm^2^, 450–490 nm, **Figure S2**) using whole field illumination for 3 s. The evoked behavior was quantified by automated video analysis and showed evoked backward movements (negative velocity) for animals expressing ChR2 in ASH and ASI. In contrast, animals that also expressed Mac in the command cells only slowed down, but did not show any reversals.

To demonstrate the utility of Arch and Mac in dissecting the functions of neurons within circuits, we analyzed the cellular pathway of nociceptive signaling downstream of the ASH neuron using these optogenetic reagents. The polymodal ASH neurons (ASH left (L) and right (R)) detect aversive osmotic [29], mechanical [20] and chemical stimuli [21]–[23] and evoke a reversal that is usually followed by a large-amplitude directional turn (called “omega” turn due to the transitional shape of the animal’s body). The neuronal circuit for this withdrawal response involves the backward command interneurons AVA, AVD and AVE to which the ASH neurons make direct synaptic contacts [11] (**Figure 5A** **, Figure S6**). ASH also makes indirect contacts (mainly via RIA), to the SMD motor neurons that are believed to assist in encoding the steep amplitude of the omega bend, and to the RIV motor neurons which specify the ventral bias of turns that follow a reversal, as indicated by laser ablation studies [30].

A variety of noxious stimuli was shown to evoke strong Ca^2+^ currents in ASH cell bodies [31], while direct electrophysiological recordings show that the DEG/ENaC channel DEG-1 is the major mechanotransduction channel in ASH to mediate nose touch [32]. Similarly, currents could be recorded from the downstream neuron AVA upon mechanical nose touch [33] and in response to photostimulating ASH neurons [34]. Photoactivation of ASH and simultaneous Ca^2+^ imaging in AVA and AVD using spatially separated light beams revealed robust responses in these downstream neurons [12]. In addition to ASH, FLP and OLQ neurons were also shown to be required for the avoidance response upon nose touch [20], [35]. Using ChR2, ASH can be photoactivated [12], [34], [36], independent of the natural stimulus and thus avoiding contributions from other neurons to the evoked behaviors. Here we assessed this small neuronal network on the behavioral level by monitoring output responses upon photoactivation of ASH and subsequent interference of downstream signaling by using Mac or Arch.

We first wanted to see if whole-field illumination can be used for activation of ChR2 in the sensory neuron of interest (ASH) and simultaneous inhibition of downstream interneurons. This application thus requires a hyperpolarizer with a blue-shifted action spectrum like Mac. Upon whole-field illumination for both activation of ChR2-expressing cells and inhibition of Mac-expressing cells, no advanced hardware setups are required, and a stereomicroscope equipped with a GFP filter would be sufficient. We generated a line expressing ChR2::YFP in ASH (and weakly in ASI) neurons by using the *sra-6* promoter. Next we introduced another extrachromosomal array containing *Pglr-1::Mac::mCherry* to achieve expression of the inhibitory proton pump Mac in the command interneurons (**Figure 5B,C**). Whole field photostimulation of worms expressing only ChR2::YFP in ASH (and ASI) with blue light (0.41 mW/mm^2^, 450–490 nm) evoked robust reversals followed by an omega turn, similar to the native avoidance response (**Video S3**). These data are in line with the observation of spontaneous Ca^2+^ increase in the backward locomotory neuron AVA when a reversal is initiated [37], [38]. In contrast, animals that in addition to ChR2 also expressed Mac in the interneurons slowed down, but did not reverse (**Video S4**). We quantified the observed behaviors by video analysis (**Figure 5D**). The obtained velocity graphs clearly illustrate the evoked backward movements (negative velocity) upon photoactivation of the sensory neurons ASH (and ASI) and the effect of photoactivated Mac as hyperpolarizer of downstream command interneurons to modulate the behavioral output by simple whole field illumination with blue light.

**Figure 6:**
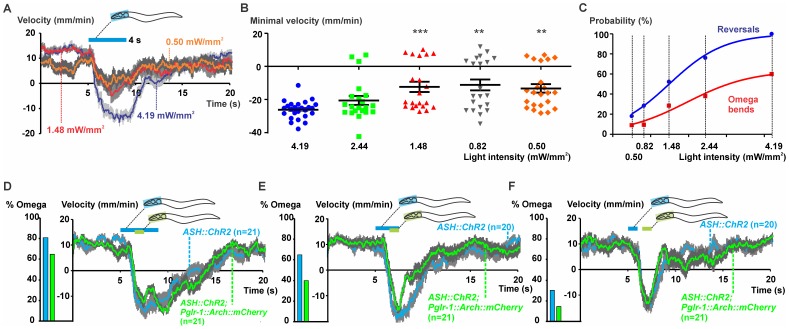
Two-color optogenetic manipulation of the ASH circuit by ChR2 and Arch. **A–C,** Head regions of animals expressing ChR2 in ASH (ASH::ChR2) were illuminated with blue (4.2 mW/mm^2^, 435–475 nm, **Figure S5**) light for 4 s using 5 different intensities. **A**, Average velocity plots are shown for three light intensities as examples. **B**, Minimal achieved velocity (highest speed when moving backward) for each individual animal was plotted; Kruskal-Wallis test followed by a Dunn’s *post-hoc* test (***p<0.0001, **p<0.005). **C**, Probabilities of the occurrence of a reversal or of an omega bend were plotted for each light intensity and resulting curves were fitted with Boltzmann sigmoidals. **D–F,** Head regions of ASH::ChR2; *Pglr-1::Arch::mCherry* animals were illuminated with blue light for **D**, 4 s, **E**, 2.5 s or **F**, 1 s. Next, the same head region was illuminated with green light (4.7 mW/mm^2^, 543–593 nm, **Figure S5**) for 1 s, 1.5 s after the start of the blue pulse. Mean velocity plots with SEMs are shown. Fractions of animals that responded with an omega bend are represented in the color-coded bar graphs.

To exclude that ASI contributed to the observed behavioral response, we next tested a strain expressing ChR2 in ASH only, by combinatorial expression via intersecting promoters and the FLP recombinase (ASH::ChR2 [36]). Illumination of the head region with blue light and thereby activating ASH only, resulted in the expected reversal behavior (**Figure 6A**). Fewer animals responded at lower light intensities and the probability of execution of an omega bend was also dependent on the light intensity, following sigmoidal relationships that could be fitted by Boltzmann functions. The behavioral dependence on light intensity was also reflected in the evoked maximal backward speed (minimal velocity) of individual animals (**Figure 6A–C**).

Next, we wanted to interfere with the behavior after the animal has initiated the reversal. To this end, two-color illumination is required to evoke the ChR2-induced backward movement with blue light and to suppress the backward movement with subsequent yellow-green illumination. To avoid inhibition of downstream signaling upon the first blue light stimulus, we further blue-shifted the stimulation light (4.2 mW/mm^2^, 435–475 nm) and supplemented the ASH::ChR2 worms with *Pglr-1::Arch::mCherry* as the more potent hyperpolarizer for yellow-green light (and less reactive in the blue range of the spectrum; **Figure 1C,D**). Illumination of the head region with blue light evoked robust reversals, which could only be suppressed by yellow-green light (1 s, 4.7 mW/mm^2^, 543–593 nm) when Arch was expressed in the downstream interneurons (**Figure 6D–F** **, Video S5**). To further investigate the dynamics of the complex behavior orchestrated by the ASH circuit (**Figure 5A** **, Figure S6**), we presented blue stimulatory pulses of different duration, but fixed the timing (1.5 s after the start of the blue light) and duration (1 s) of the inhibiting light pulses. First, we observed that the length of the backward movement and the probability of the occurrence of an omega bend were also dependent on the length of the photostimulus. 80% of the ASH::ChR2 animals responded with a long reversal followed by an omega bend when using a 4 s blue light pulse. A concomitant 1 s yellow-green light pulse after 1.5 s induced a short interruption of the backward movement when Arch was expressed in the command interneurons. Nevertheless, after this forced hyperpolarization, the backward movement continued, as if the command cells continued to receive signals from ASH; furthermore, 65% of the animals still executed an omega turn (**Figure 6D**). When shortening the blue light pulse to 2.5 s (**Figure 6E**) or 1 s (**Figure 6F**), fewer animals responded with an omega bend (e.g. only 30% of the worms showed omega bends when challenged with a blue light pulse of 1 s; **Figure 6F**). Also, the achieved negative speeds were reduced when compared to the 4 s stimulation.

These results indicate that the length and intensity of the stimulating blue light pulse can be modulated to trigger varying behavioral outputs. Prolonged activation of ASH results in a longer reversal and a higher probability of executing a directional turn, indicating that this neuron is capable of continued activation of downstream neurons. Combining different multi-color illumination protocols with the outward directed proton pumps Mac and Arch as new and powerful neuronal hyperpolarizers enabled us to investigate temporal aspects of signaling downstream of ASH.

## Discussion

We compared the currently available optogenetic hyperpolarizers in *C. elegans* and found that both Mac and Arch are more efficient when compared to NpHR. In addition, the action spectrum of Mac is more blue-shifted compared to Arch and NpHR. This opens the possibility to stimulate neurons under study *via* ChR2 and simultaneously evoke neuronal silencing of downstream neurons with Mac using blue light, as we showed for the ASH circuit. Potentially, additional neuronal populations can be silenced by Arch or NpHR with yellow or red light, due to the different spectral characteristics of the three hyperpolarizers, and using the multimodal illumination and tracking system we described previously [6], [19]. Here, we demonstrated the utility of Arch and Mac as inhibitors or “circuit breakers” in two neuronal networks for mechanosensation and nociception, concomitant with optical activation of upstream neurons *via* ChR2 by blue light.

### Arch and Mac as Potent Hyperpolarizers to Complement the Optogenetics Toolbox

Neuronal activity can be inhibited using halorhodopsin from *Natronomonas pharaonis* (NpHR) and yellow light. However, in *C. elegans*, NpHR often is insufficient to robustly inhibit individual neurons, likely due to poor trafficking to the plasma membrane. Thus, effective NpHR application is possible only if a strong promoter is available for the cell of interest. Versions of NpHR with better trafficking have been generated [17], [18]. However, various signal or trafficking sequences attached to NpHR did not improve trafficking in *C. elegans*. Thus, we explored the use of other (more potent) rhodopsin-type hyperpolarizers, such as the outward directed proton pumps Arch and Mac [8]. As cells and extracellular fluid are strongly buffered, shuffling protons across the membrane is not expected to cause any appreciable pH changes. The action spectra of these new optogenetic tools were reported to be more blue-shifted than NpHR (primary peaks of their action spectra are 566 nm for Arch and 550 nm for Mac, vs. 590 nm for NpHR).

Using behavioral assays and current recordings on dissected muscle cells expressing NpHR, Arch or Mac, we show here that the yellow-green light driven proton pump Arch provides larger outward currents than NpHR, even with illumination of 590 nm yellow light, while the transgenic muscle cells exhibited similar input resistances. The green light-driven hyperpolarizing proton pump Mac can be triggered with the same yellow light, as well as with blue light of 470 nm. To investigate if these new tools would be useful for neuronal inhibition, we expressed Arch and Mac in the cholinergic neurons. In agreement with previous results for NpHR [7], illumination of worms expressing Mac with yellow-green light caused the nematodes to stop their forward movement, for all hyperpolarizers. With blue light, the locomotory blockade was less pronounced when Arch was expressed, whereas no obvious change in velocity could be observed when using NpHR.

Combined with multimodal illumination strategies, Arch and Mac are highly useful for interfering with downstream signaling in networks in which upstream neurons are stimulated by ChR2. This was demonstrated by interfering with downstream signaling from the touch receptor neurons. Photoactivation of AVM and ALML/R resulted in robust reversals. A behavioral switch to forward locomotion could abruptly be induced by photoinhibition of *Pglr-1::Arch-*expressing neurons in the head. Though these results clearly show that we can interfere with downstream signaling of sensory neurons, it is unclear to interpret these observations in terms of *in vivo* cell physiology, also as the *glr-1* promoter used to express the hyperpolarizers is active not only in the command neurons. Yet, with some caution, the observed blocking of an initiated reversal and transition to forward movement can be interpreted as an acute stopping (hyperpolarization) of highly active (backward) command neurons, thereby blocking the ability to receive input signals from the sensory cells AVM and ALML/R. In addition, hyperpolarization of the command cells is also likely to inhibit or block output signaling to the backward motor neurons. Furthermore, this selective head neuron hyperpolarization spares one of the forward command neurons, PVC (situated in the tail), and may thus induce an imbalance in the command neuron network that could result in forward locomotion. However, no accelerated forward movement was induced when inhibiting the command interneurons in the head in animals that already crawl forward (no ChR2 was expressed in these control experiments). In sum, we showed that using Arch one can clearly interfere with downstream signaling form (photoactivated) sensory neurons and thus conclude that Arch is the hyperpolarizer of choice when using two-color optogenetics to dissect a neuronal circuit.

### Dissection of the Nociceptive ASH Network

ASH neurons sense diverse chemical stimuli [21]–[23], osmotic stress [29] and mechanical stimuli to the nose [20]. These diverse insults evoke withdrawal behaviors, involving direct synaptic contacts from ASH to the backward command interneurons AVA, AVD and AVE [11]. However, the circuitry involving ASH is complex, making it difficult to functionally dissect it. For example, ASH forms synapses also to the forward command interneuron AVB, however, as AVA and AVD together receive more synapses from ASH, the backward command is likely predominant, or neurotransmitters released from ASH to AVB act inhibitory. ASH is also (indirectly) connected to the motor −/interneurons SMD and RIV that are mainly responsible for the execution of the omega bend, as deduced from cell ablation experiments [30]. Acute activation and inhibition by light adds a more versatile approach to the analysis of the behavioral output of a circuit, which can clearly not be deduced simply by analyzing synaptic wiring. It is particularly difficult to study the output behavior of ASH as roughly 10% of the 302 neurons in *C. elegans* (32 neurons) are believed to be chemosensory [39], and so other cells could contribute to the effects of particular chemicals. Furthermore, FLP and OLQ neurons in the head also contribute to nose-touch sensation [20], [35]. Using optogenetics, however, one can specifically photoactivate ASH by ChR2 [12], [34], [36], independent of the various natural stimuli and without concomitant activation of other contributing neurons.

Here, we used the ASH circuit as an example for functional dissection by using several optogenetic tools and spatially selective illumination procedures, altering color, duration and intensity of the light. We found that blocking downstream signaling by Mac and Arch in command interneurons efficiently inhibited ChR2-evoked reversal behaviors. Photoactivation of ASH using ChR2 evoked robust reversals, often followed by an omega bend. This output behavior was similar to the observed behavioral output for natural stimuli. The probability of a reversal and omega bend depended on light intensity. Assuming graded signals in ASH, this should translate into amount of transmitter released, and consistently, this followed Boltzmann sigmoidal relations. Omega bend probability further depended on stimulus duration. Blue light pulses of 1 s could only evoke omega bends in 30% of the animals tested, although nearly all animals responded with a (brief) backward movement. In contrast, long reversals followed by an omega bend were induced in 80% of the animals when photostimulating ASH for 4 s.

When using a simple hardware setup allowing illumination with one color (blue) at the same time, we showed that photoactivation of the sensory neurons ASH (and ASI) and simultaneous photohyperpolarization of the *Pglr-1::Mac-*expressing (command) neurons for a few seconds, slowed down the animals and blocked or inhibited the locomotory program. This way, the intended backward movement resulting from photodepolarization of ASH (and ASI) was blocked from the beginning. To show that one can also interfere with an initiated motor program, we used a more advanced setup allowing timed illuminations of different body segments with different colors [6]. Simultaneous inhibition of the command cells in the head expressing Arch using a yellow-green light pulse of 1 s, 1.5 s after the onset of the blue pulse, temporarily interrupted the initiated backward movement. The command cells thus are likely to receive continued synaptic inputs from ASH and the reversal is continued during the last part of the blue light pulse.

Taken together, our data show that ASH continues to signal to *glr-1*-expressing neurons during extended stimulation and that output from ASH is graded. This logic is appropriate for a nociceptive neuron, which should continue to signal the threatening stimulus as long as it can be detected. Our findings are in line with previous imaging experiments showing that increased Ca^2+^ levels in ASH are maintained throughout persistent stimuli [31]. Whole-cell patch clamp recordings from ASH revealed increased amplitudes of mechanoreceptor currents when more force was applied to the nose tip [32], showing that the graded output response downstream from ASH is occurring on the level of the sensory neuron itself. When photoactivating ASH while imaging Ca^2+^ transients in downstream neurons, Guo and colleagues found that Ca^2+^ levels in AVA and AVD increased a few seconds after the start of the photostimulus for ASH, and declined a few seconds after the end of the light pulse [12]. These data are generally in line with our behavioral results, where we also observed a small delay of the evoked backward movement, relative to the start of the activating light pulse. However, Guo et al. used the GABA_A_ receptor agonist muscimol to immobilize animals for imaging, which may have slowed down signaling further. When assessing Ca^2+^ dynamics in ASH, however, a clear synchronization of [Ca^2+^] increases with the stimulating light pulse was shown [12], indicating that the behavioral delay is evoked on the level of downstream signaling. Circuit analyses combining optogenetic tools, i.e. ChR2, Arch and Mac, with selective illumination in behavioral assays, and, ideally Ca^2+^ imaging, will be a major approach to understanding nervous system function in *C. elegans*. Improved tools like red-shifted channelrhodopsins or Ca^2+^ sensors are emerging and similar multi-spectral illuminators will be increasingly common and increasingly easy to implement in the near future.

## Materials and Methods

### Molecular Biology

To generate pCS10 (*Pmyo-3::NpHR-SigSeq::NpHR::eCFP*), a 57 bp fragment corresponding to the native signal sequence of NpHR was inserted into AgeI and XbaI sites of the *Pmyo-3::NpHR::eCFP* plasmid [7]. The insert was created by annealing two pairs of primers: oCS30 (CTAGAATGCGACTAGTCGGTATCCGTTTGATTTC), oCS33 (TAGAGGAATGAAATCAAACGGATACCGACTAGTCGCATT), and oCS31 (ATTCCTCTACGTAGTATATGATGGAGCCATGACTGAGACATTGCCA), oCS32 (CCGGTGGCAATGTCTCAGTCATGGCTCCATCATATACTACG).

For pCS30 (*Pmyo-3::eNpHR 2.0(human codon optimized)::eYFP*), we amplified *eNpHR 2.0(human codon optimized)::eYFP* from a plasmid obtained from K. Deisseroth using oCS84 (CAGGATCCATGAGGGGTACGCCCCTG) and oCS85 (AGGAATTCTTACACCTCGTTCTCG), and swapped this insert with an EcoRI-BamHI-flanked *ChR2::YFP* from a *Pmyo-3::ChR2::YFP* plasmid [5].

Generation of pCS31 (*Pmyo-3::NpHR(human codon optimized)::eYFP*): A fragment containing *NpHR::eYFP (eNpHR without nAChR-signal sequence and ER-export motif)* was amplified from a plasmid containing eNpHR 2.0 (provided by K. Deisseroth [17]) by PCR using the primers oCS86 (CAGGATCCATGACAGAGACCCTGCCTCCC) and oCS87 (AGGAATTCTTACTTGTACAGCTCGTCC) and ligated into the *Pmyo-3*-containing backbone.

pCS53 (*Pmyo-3::eNpHR 2.0(human codon optimized; not including the nAChR-motif)::eYFP*): A fragment containing *eNpHR::eYFP (eNpHR without nAChR-signal sequence)* was amplified from pCS30 using oCS86 (CAGGATCCATGACAGAGACCCTGCCTCCC) and oCS85 (AGGAATTCTTACACCTCGTTCTCG) and cloned into the *Pmyo-3*-containing backbone.

Generation of pCS71 (*Pmyo-3::NpHR::eCFP::dop-1 ER export motif*) and pCS72 (*Pmyo-3::NpHR-SigSeq::NpHR::eCFP::dop-1 ER export motif*): Two fragments were amplified by PCR from pCS10 using the primers oMB42 (GGATACAGCTTCCTCGACATCG) and oCS136 (CTTCTTGAATGCACGACGGAAATCACGATTGAACTTGTACAGCTCGTCCATGCCGAG), as well as oCS151 (ACGGTACCGCTTATTTCATTTCCAAG), and oCS135 (TTCAATCGTGATTTCCGTCGTGCATTCAAGAAGTAAAGCGGCCAATTCCAACTGAG). After digestion with BsmI, both fragments were used in a PCR reaction as template with primers oMB42 and oCS151. The resulting product was then cloned in *Pmyo-3::NpHR::eCFP* and pCS10, respectively, using the restriction enzymes NcoI and BsiWI to result in pCS71 and pCS72.

pCS76 (*Pmyo-3::NpHR(dop-1 ER export motif after Ser272)::eCFP)* and pCS77 (*Pmyo-3::NpHR-SigSeq::NpHR(dop-1 ER export motif after Ser272)::eCFP*): Two fragments were amplified by PCR from pCS10 using the primers oCS40 (CCATCTAGAATGACTGAGAC) and oCS133 (CAGAATGCACGACGGAAATCACGATTGAAGCTCTCGTTCGACGTGAGG), as well as oCS151 (ACGGTACCGCTTATTTCATTTCCAAG) and oCS134 (GTTGCATTCAAGAAGGTCGTCTCCGGCTCGATACTCG). After digestion with BsmI, both fragments were used in a PCR reaction as template with the primers oCS40 and oCS151. The resulting product was then cloned into *Pmyo-3::NpHR::eCFP* and pCS10, respectively, using the restriction enzymes AccI and BsiWI to result in pCS76 and pCS77.

Arch and Mac plasmids: *Arch::GFP* was obtained as a BamHI-EcoRI fragment from the *FKC-Arch::GFP* plasmid [8] and swapped with the *ChR2::YFP* fragment from the *Pmyo-3::ChR2::YFP* vector to obtain pSH120 (*Pmyo-3::Arch::GFP*). Analogously, *Mac::GFP* was subcloned from *FKC-Mac::GFP* into the *Pmyo-3* expression vector to generate pSH121 (*Pmyo-3::Mac::GFP*). In order to generate pSH122 (*Punc-17::Arch::YFP*), *Arch* was amplified by PCR using Arch_NheI_F (ATCTAGCTAGCATGGACCCCATCGCTCTGC) and Arch_KpnI_R (GGTACCCGGTCGGCGGCACTGACAT) and ligated into the *Punc-17::ChR2::YFP* backbone from which *ChR2* was removed. Similarly, *Mac* was amplified with primers Mac_NheI_F (ATCTAGCTAGCATGATCGTGGACCAGTTCG) and Mac_KpnI_R (GGTACCCGGGCGCCGTCGTCCTCG) and subcloned in the same backbone to obtain pSH123 (*Punc-17::Mac::YFP*). We ligated the BamHI-AgeI-flanked *Arch* or *Mac* into pNP165 *Pglr-1::floxStopfloxChR2::mCherry* in which the floxStopfloxChR2 fragment was removed to generate pSH127 (*Pglr-1::Arch::mCherry*) and pSH128 (*Pglr-1::Mac::mCherry*).

### Strains

All *C. elegans* strains were cultivated at 20°C on standard nematode growth medium (NGM) and fed with *E. coli* OP50-1 bacteria. Genotypes and transgenes of all strains used in this work are listed below:

**ZX444**: *lin-15(n765ts; zxEx301[Pmyo-3::NpHR::eCFP; lin-15+]*, **ZX619**: *lin-15(n765ts); zxEx492[Pmyo-3::eNpHR 2.0 (human codon optimized)::eYFP; lin-15+]*, **ZX711**: *lin-15(n765ts); zxEx420[Pmyo-3::NpHR-SigSeq::NpHR::eCFP::dop-1 ER export motif; lin-15+]*, **ZX830**: *lite-1(ce314); zxEx620[Punc-17::Mac::YFP; Pelt-2::mCherry]*, **ZX831**: *lite-1(ce314); lin-15(n765ts); zxEx700[Psra-6::ChR2::YFP; lin-15+]*, **ZX896**: *lite-1(ce314); ljIs123[Pmec-4::ChR2; Punc-122::RFP]; zxEx436[Pglr-1::NpHR::eCFP; Pelt-2::mCherry]*; **ZX899**: *lite-1(ce314); ljIs123[Pmec-4::ChR2; Punc-122::RFP]; zxEx621[Pglr-1::Mac::mCherry; Pelt-2::GFP]*, **ZX901**: *lite-1(ce314); zxEx623[Punc-17::Arch::YFP; Pelt-2::mCherry]*, **ZX936**: *lite-1(ce314); zxEx629[Pmyo-3::Mac::GFP; Pelt-2::mCherry]*, **ZX937**: *lite-1(ce314); zxEx630[glr-1::Arch::mCherry; Pelt-2::GFP]*, **ZX938**: *lite-1(ce314); zxEx631[glr-1::Mac::mCherry; Pelt-2::GFP]*, **ZX972**: *lite-1(ce314); lin-15(n765ts); zxEx700[Psra-6::ChR2::YFP; lin-15+]; zxEx621[Pglr-1::Mac::mCherry; Pelt-2::GFP]*, **ZX1024**: *lite-1(ce314); lin-15(n765ts); zxEx700[Psra-6::ChR2::YFP; lin-15+]; zxEx636[Pglr-1::Arch::mCherry; Pelt-2::GFP]*, **ZX1026**: *lite-1(ce314); zxEx628[Pmyo-3::Arch::GFP; Pelt-2::mCherry]*, **ZX1029**: *lite-1(ce314); ljIs123[Pmec-4::ChR2; Punc-122::RFP]; zxEX636[Pglr-1::Arch::mCherry; Pelt-2::GFP]*, **ZX1124**: *lite-1(ce314); ljIs114[Pgpa-13::FLPase; Psra-6::FTF::ChR2::YFP]*; *zxEX636[Pglr-1::Arch::mCherry; Pelt-2::GFP]*, **ZX1126**: *lite-1(ce314); ljIs114[Pgpa-13::FLPase; Psra-6::FTF::ChR2::YFP]; zxEx621[Pglr-1::Mac::mCherry; Pelt-2::GFP]*. **AQ2334**: *lite-1(ce314); ljIs123[Pmec-4::ChR2; Punc-122::RFP]*, **AQ2235**: *lite-1(ce314); ljIs114[Pgpa-13::FLPase; Psra-6::FTF::ChR2::YFP]*.

### Confocal Microscopy

Young adult animals were immobilized on 3% agarose pads in M9 buffer that was supplemented with 30 mM NaN_3_. Z-stacks were recorded using a Zeiss LSM 510 laser scanning microscope equipped with a Plan-Apochromat 63×/1.4 oil DIC objective. CFP, GFP or mCherry were excited at 405 nm, 488 nm or 543 nm respectively and fluorescence was monitored using BP420–480, BP505–530 or LP560 filters. Projections of the Z-stacks were generated and overlaid using ImageJ software (NIH).

### Optogenetics

The *E. coli* OP50 culture that was used to grow a bacterial lawn on the NGM plates was supplemented with all-*trans* retinal (ATR, Sigma-Aldrich) to a final concentration of 100 µM. Animals for optogenetic analyses were cultivated on these plates at least for one generation at 20°C. At least 30 minutes prior to behavioral analysis, young adult animals were transferred to new NGM plates without OP50 bacteria. For all optgenetic experiments, light intensity was monitored using an optical power meter (PM100, Thorlabs).

Analysis of relaxation effects induced by NpHR, eNpHR 2.0, and NpHR-variants including motifs to promote cell-surface expression was performed as described earlier [7]. Briefly, animals were transferred to 5,5 cm dishes containing 4 ml of nematode growth medium (NGM) and recorded on an Axiovert 40 CFL microscope (Zeiss) with 10x magnification using a Powershot G9 digital camera (Canon). For photoactivation, yellow light from an HBO50 light source (530–560 nm; filter F41-007, AHF Analysetechnik) was presented and controlled by a computer-driven shutter (Sutter Instruments). Light intensities were adjusted using neutral density filters (AHF Analysetechnik). Videos were extracted into single frames and analyzed using custom scripts for Matlab or ImageJ.

For speed analysis during whole field illumination, we used a stereomicroscope (Leica, MZ16F) equipped with an external fluorescence light source with built-in shutter. Blue light was obtained by using a GFP3 filter (450–490 nm, Leica), and yellow-green light (540–580 nm) was obtained using a mCherry filter (**Figure S1**). The electronic shutter and a digital camera (Sony XCD-SX90, operated at 15 fps) was controlled by a custom MATLAB (The MathWorks Inc.) script, which is available at http://www.biochem.uni-frankfurt.de/index.php?id=236. We used the parallel worm tracker [25] as a video analysis tool to obtain speed data for all individual animals.

The relative body length of the animals was calculated by computer-driven analysis of the worm shapes for individual video frames, as described earlier [3]. We used an inverted epi-fluorescence microscope (Axiovert 200, Zeiss) equipped with a HBO100 light source along with 10 20 nm band-pass filters (Edmund Optics; 400, 420, 458, 480, 500, 520, 540, 568, 580 and 600 nm, **Figure S2**) in order to measure the action spectra of NpHR, Mac and Arch. A digital USB camera (DCC1545M, Thorlabs, operated at 10 fps) and an electronic shutter (Sutter Instruments) were controlled via a custom program written in LabView. Activity spectra for each hyperpolarizer were generated by plotting the normalized (maximal elongation is set to 1 for each strain) average body elongations for each wavelength, calculated for a 1.5 s time frame, starting 0.5 s after the onset of the light until the end of the light pulse.

For the “selective illumination and tracking” setup, we equipped an inverted epi-fluorescence microscope (Axiovert 35, Zeiss) that allows tracking of the freely behaving worm with a commercially available LCD projector (Hitachi CP-X605) as described previously [6], [19]. In short, live images were acquired with a digital USB camera (DCC1545M, Thorlabs) and the worm under study was tracked by a computer-controlled x-,y-translational stage. Different segments of the nematode could be illuminated by sending the appropriate illumination pattern to the projector essentially in real-time. A 475 nm short-pass filter (UQG optics) was added in the blue path, a 568/50 nm filter (Chroma) in the green path, and a 675 nm short-pass filter (UQG Optics) in the red path of the three-color LCD projector (**Figure S4**).

### Electrophysiology

Whole-cell voltage clamp recordings (holding potential was −40 mV) on dissected *C. elegans* body wall muscle cells were performed as described previously [5]. Light activation was performed using an LED lamp (KSL-70, Rapp OptoElectronic, Germany) at a wavelength of 590 nm (3.82 mW/mm^2^) or 470 nm (8.06 mW/mm^2^), and controlled by the HEKA software (**Figure S3**). Where appropriate, the light intensity of the LED lamp was reduced using the control unit.

### Statistics

To test whether datasets for statistical analysis follow Gaussian distributions, we performed D’Agostino and Pearson omnibus normality tests. Statistical differences were tested using Student’s t tests or one-way ANOVA followed by Dunnett’s *post-hoc* tests, as indicated in the figure legends. A non-parametric Kruskal-Wallis test followed by a Dunn’s *post-hoc* test was performed when the data did not follow a Gaussian distribution. To compare average velocities of animals from the same genotype at different time points (before, during and after a light pulse), we used a non-parametric repeated measures test (Friedman) as the velocities of the individual animals were not normally distributed, followed by a Dunn’s multiple comparison test. As displayed in the figure legends, all error bars represent SEMs.

## Supporting Information

Figure S1 **Efficiency of modified NpHR-variants for optogenetic hyperpolarization of muscle cells.** **A**, Scheme depicting modifications applied to NpHR to enhance cell surface expression. For WT-NpHR, a motif of 19 amino acids resulting from a 57 bp region upstream of the start-codon of NpHR from the genome of *Natronomonas pharaonis*, was added to the N-terminus to result in a putative eukaryotic signal sequence (termed “native signal sequence”). We also inserted a conserved ER export motif from DOP-1 (N‘-FNRDFRRAF-C’; also see [24]) directly after TM7 (after Ser272 in NpHR) or after eCFP. For human codon-optimized NpHR, an ER exit motif from the Kir2.1 inward rectifying potassium channel was added after eYFP at the C-terminus and a signal peptide from an nAChR (beta-subunit) was included at the N-terminus (resulting in eNpHR2.0 [17]). **B**, Various NpHR-variants as depicted in (**A**) were expressed in BWMs while the resulting relaxation effects, i.e. increase in normalized body length were measured upon yellow light photoactivation (530–590 nm; 10 mW/mm^2^); mean values are indicated with SEMs; n = 11–20. Statistical analysis was performed using ANOVA followed by Dunnett’s multiple comparison test; ***p<0.0001, **p<0.005, *p<0.05, n.s. not significant. **C,** Relaxations induced either by WT-NpHR or WT-NpHR with additional N-terminal native signal sequence and C-terminal DOP-1 ER export motif were further analyzed at different light intensities (530–590 nm; 0.02–10 mW/mm^2^); mean values are shown with SEMs; n = 10–18.
(TIF)

Figure S2 **Spectra of blue and yellow-green light for whole-field illumination.** Blue light for whole-field illumination was obtained by using a GFP3 filter (450–490 nm, Leica), and yellow-green light (540–580 nm) was obtained using a mCherry filter. Resulting spectra are shown.
(TIF)

Figure S3 **Spectra from all band-pass filters used.** In order to measure the action spectra of NpHR, Mac and Arch, we used an inverted epi-fluorescence microscope (Axiovert 200, Zeiss) equipped with a HBO100 light source and 10 20 nm band-pass filters (Edmund Optics; 400, 420, 458, 480, 500, 520, 540, 568, 580 and 600 nm). Resulting spectral output is shown for each filter.
(TIF)

Figure S4 **Spectra from 470nm LED and 590nm LED light sources.** Light activation for patch-clamp recordings from dissected *C. elegans* body muscles was performed using LED lamps (KSL-70, Rapp OptoElectronic, Germany) at a wavelength of 470 nm or 590 nm. The spectra ofthe light sources used are shown.
(TIF)

Figure S5 **Spectra from blue and green light sources used for selective illumination.** Different segments of a freely moving worm could be illuminated by sending the appropriate illumination pattern to a modified video projector that was aligned with the epi-fluorescence port of an inverted microscope (Axiovert 35, Zeiss). A 475 nm short-pass filter (UQG optics) was added in the blue path and a 568/50 nm filter (Chroma) in the green path; resulting spectra are shown.
(TIF)

Figure S6 **Wiring diagram of the polymodal nociceptive neuron ASH.** Schematic representation of the wiring diagram of the ASH circuit with indication of the synaptic contacts and gap junctions according to [11].
(TIF)

Video S1 **Analysis of** ***C. elegans*** **body length when hyperpolarizing body wall muscle cells using Mac.** We challenged animals expressing Mac in body wall muscle cells (ZX936: *lite-1(ce314); zxEx629[Pmyo-3::Mac::GFP; Pelt-2::mCherry]*) with 10 yellow-green (0.96 mW/mm^2^, 540–580 nm) light pulses of 1.5 s using whole-field illumination, with an interstimulus interval (ISI) of 1.5 s and calculated the percentage of length increase by automated video analysis. Related to Figure 1.
(MOV)

Video S2 **Photoinhibition of cholinergic neurons by Mac temporarily reduces crawling speed.** We expressed Mac in the cholinergic neurons using the promoter of the *unc-17* gene (ZX830: *lite-1(ce314); zxEx620[Punc-17::Mac::YFP; Pelt-2::mCherry]*) and analyzed the velocity of the animal by video analysis. A yellow-green (0.96 mW/mm^2^, 540–580 nm) light pulse of 1 s causes the nematode to temporarily interrupt its forward locomotion. Related to Figure 3.
(MOV)

Video S3 **Photoactivation of ASH by whole-field illumination evokes backward movements and omega bends.** We expressed ChR2 in ASH (and ASI) neurons using the *sra-6* promoter (ZX831: *lite-1(ce314); lin-15(n765ts); zxEx700[Psra-6::ChR2::YFP; lin-15+])* and photoactivated these sensory neurons using whole-field illumination with a blue light pulse (0.41 mW/mm^2^, 450–490 nm) of 3 s, causing the animals to reverse (negative velocity). In addition, a directional turn, called “Omega bend” is executed. Related to Figure 5.
(MOV)

Video S4 **Simultaneous photoactivation of ASH and inhibition of downstream neurons** ***via*** **Mac using whole-field illumination.** ZX831 animals were modified with an additional extrachromosomal array containing *Pglr-1::Mac::mCherry* to achieve expression of Mac in downstream command interneurons (and other cells; ZX972: *lite-1(ce314); lin-15(n765ts); zxEx700[Psra-6::ChR2::YFP; lin-15+]; zxEx621[Pglr-1::Mac::mCherry; Pelt-2::GFP]*). Using whole-field illumination with a blue light pulse (0.41 mW/mm^2^, 450–490 nm) of 3 s, we photoactivated ASH and ASI neurons and simultaneously inhibited downstream signaling, causing the animals to slow down upon illumination; no backward movement was observed. Related to Figure 5.
(MOV)

Video S5 **Multimodal illumination allows photoactivation of ASH and inhibition of downstream signaling** ***via*** **Arch.** Using multimodal illumination, ASH was photoactivated by projecting blue light (4.2 mW/mm^2^, 435–475 nm) on the head of the animals, evoking a reversal. This backward movement could temporarily be interrupted by inhibition of downstream command interneuron *via* Arch and illumination of the head region with green light (4.7 mW/mm^2^, 543–593 nm). Backward movement is continued after the inhibitory light pulse ended and an omega bend is executed. Related to Figure 6.
(MOV)
