# Supplementary figures and images for: Microbial Light-Activatable Proton Pumps as Neuronal Inhibitors to Functionally Dissect Neuronal Networks in C. elegans
Source: PLoS One. 2012 Jul 16;7(7):e40937. doi: 10.1371/journal.pone.0040937 (PMC3397962; doi:10.1371/journal.pone.0040937)

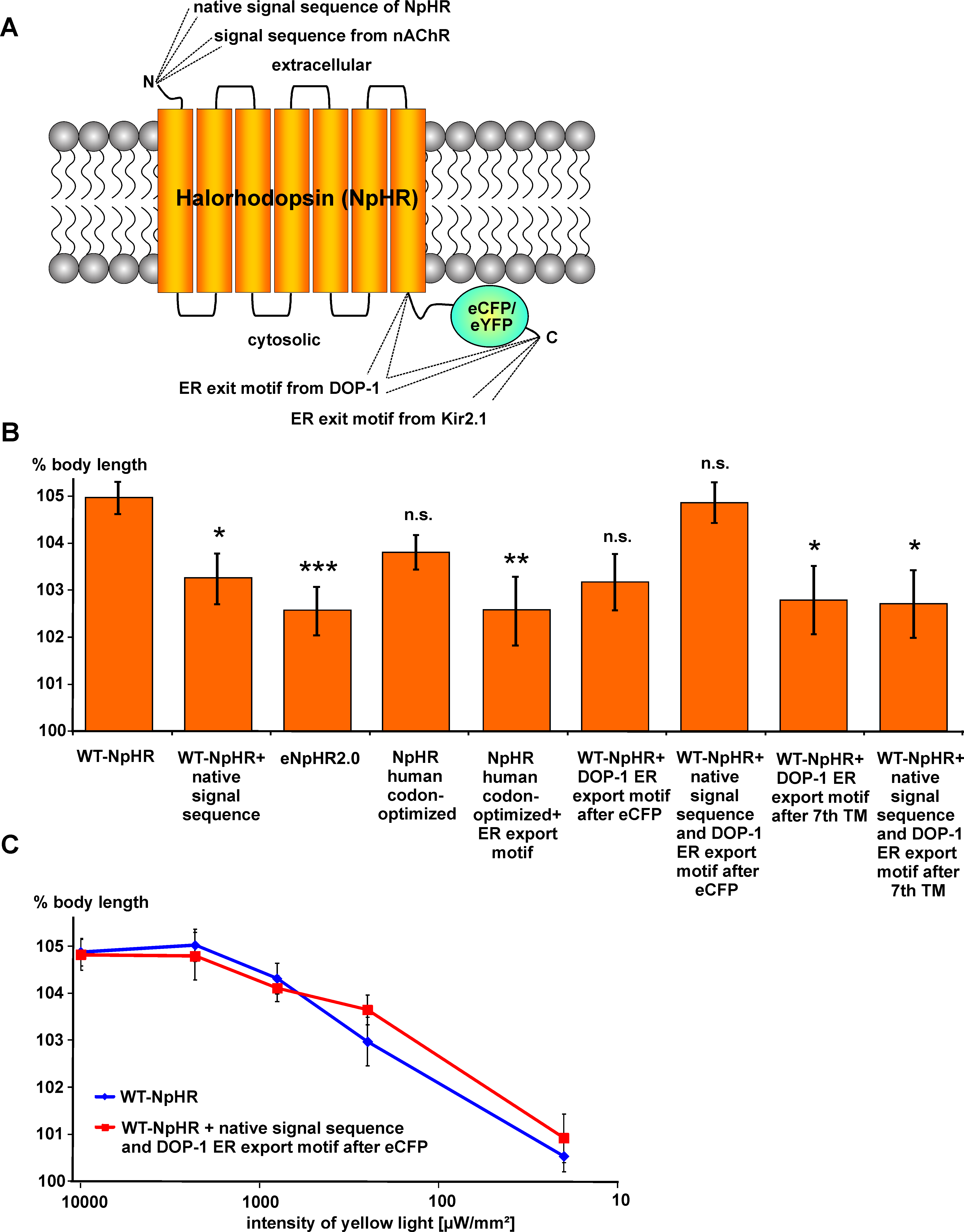

Supplement: Figure S1 — Efficiency of modified NpHR-variants for optogenetic hyperpolarization of muscle cells. A, Scheme depicting modifications applied to NpHR to enhance cell surface expression. For WT-NpHR, a motif of 19 amino acids resulting from a 57 bp region upstream of the start-codon of NpHR from the genome of Natronomonas pharaonis, was added to the N-terminus to result in a putative eukaryotic signal sequence (termed “native signal sequence”). We also inserted a conserved ER export motif from DOP-1 (N‘-FNRDFRRAF-C’; also see [24]) directly after TM7 (after Ser272 in NpHR) or after eCFP. For human codon-optimized NpHR, an ER exit motif from the Kir2.1 inward rectifying potassium channel was added after eYFP at the C-terminus and a signal peptide from an nAChR (beta-subunit) was included at the N-terminus (resulting in eNpHR2.0 [17]). B, Various NpHR-variants as depicted in (A) were expressed in BWMs while the resulting relaxation effects, i.e. increase in normalized body length were measured upon yellow light photoactivation (530–590 nm; 10 mW/mm2); mean values are indicated with SEMs; n = 11–20. Statistical analysis was performed using ANOVA followed by Dunnett’s multiple comparison test; ***p<0.0001, **p<0.005, *p<0.05, n.s. not significant. C, Relaxations induced either by WT-NpHR or WT-NpHR with additional N-terminal native signal sequence and C-terminal DOP-1 ER export motif were further analyzed at different light intensities (530–590 nm; 0.02–10 mW/mm2); mean values are shown with SEMs; n = 10–18. (TIF) [file pone.0040937.s001.tif]

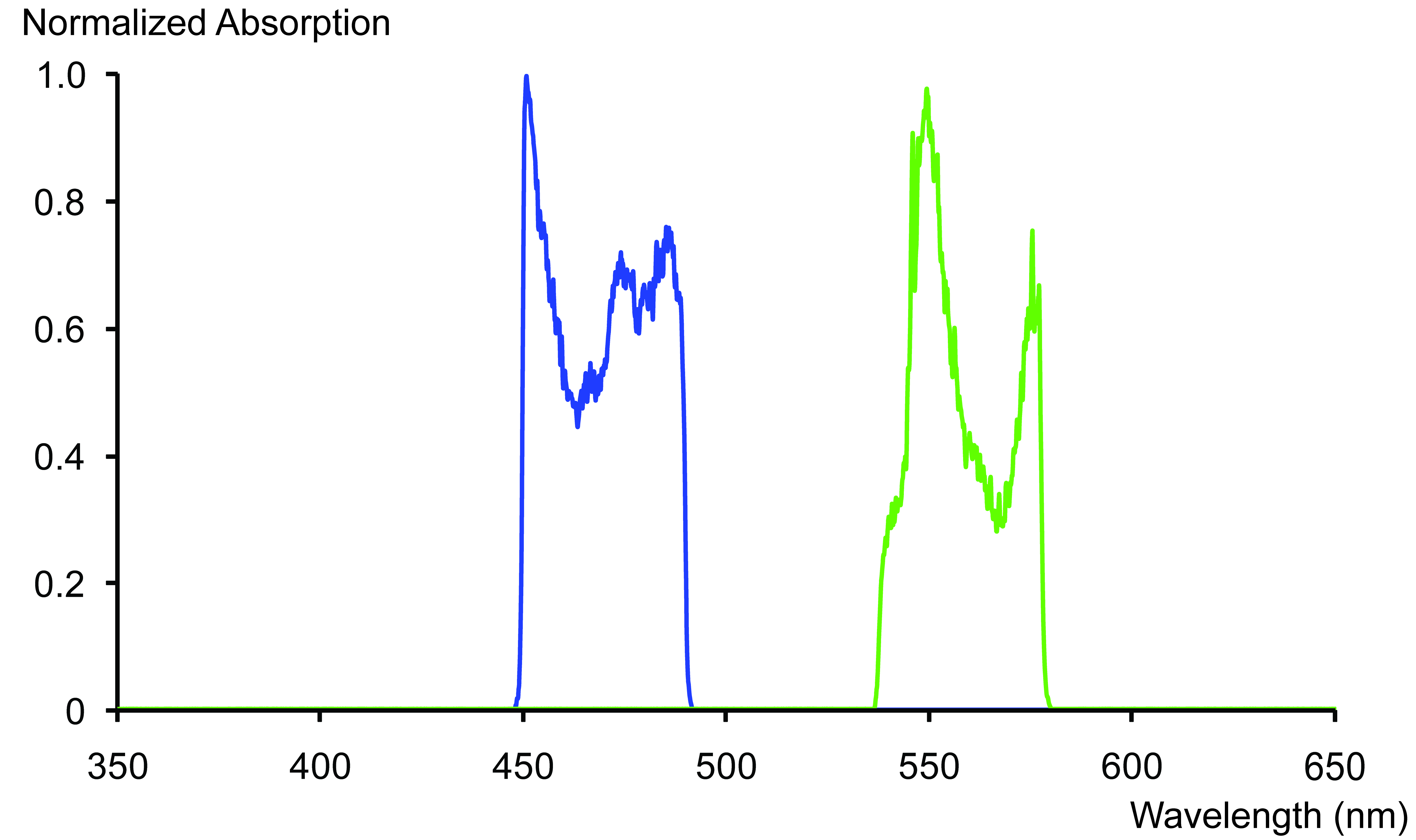

Supplement: Figure S2 — Spectra of blue and yellow-green light for whole-field illumination. Blue light for whole-field illumination was obtained by using a GFP3 filter (450–490 nm, Leica), and yellow-green light (540–580 nm) was obtained using a mCherry filter. Resulting spectra are shown. (TIF) [file pone.0040937.s002.tif]

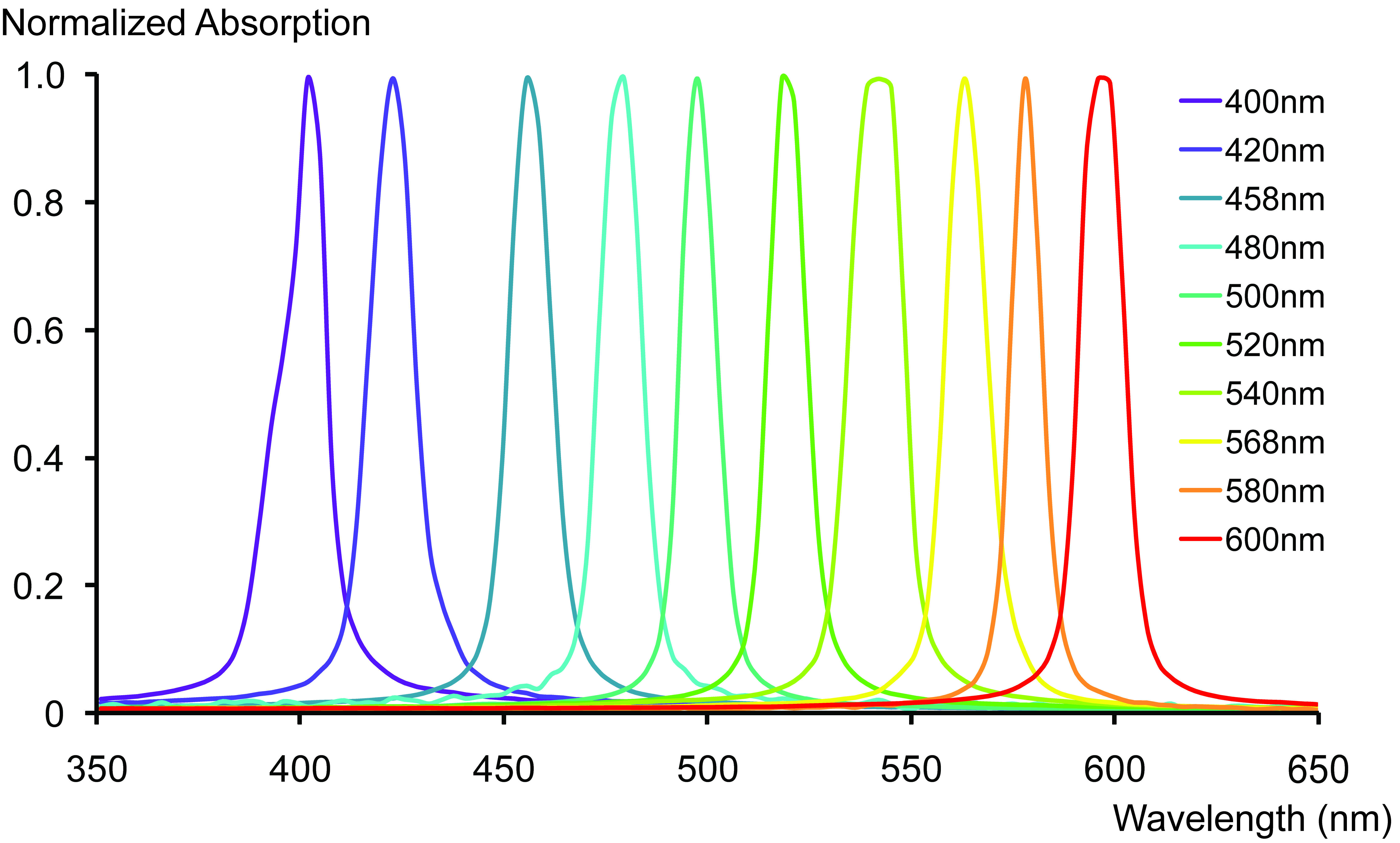

Supplement: Figure S3 — Spectra from all band-pass filters used. In order to measure the action spectra of NpHR, Mac and Arch, we used an inverted epi-fluorescence microscope (Axiovert 200, Zeiss) equipped with a HBO100 light source and 10 20 nm band-pass filters (Edmund Optics; 400, 420, 458, 480, 500, 520, 540, 568, 580 and 600 nm). Resulting spectral output is shown for each filter. (TIF) [file pone.0040937.s003.tif]

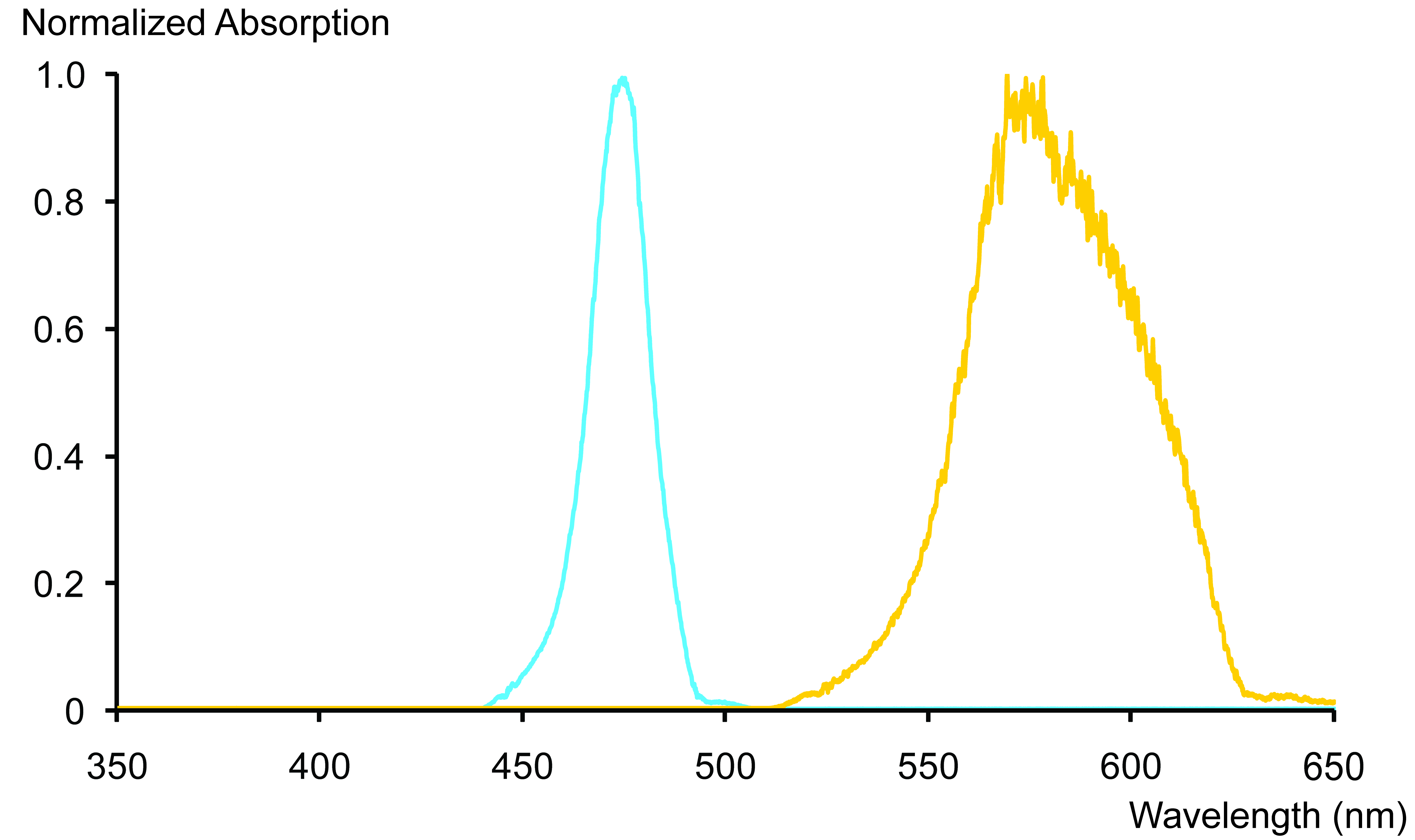

Supplement: Figure S4 — Spectra from 470nm LED and 590nm LED light sources. Light activation for patch-clamp recordings from dissected C. elegans body muscles was performed using LED lamps (KSL-70, Rapp OptoElectronic, Germany) at a wavelength of 470 nm or 590 nm. The spectra ofthe light sources used are shown. (TIF) [file pone.0040937.s004.tif]

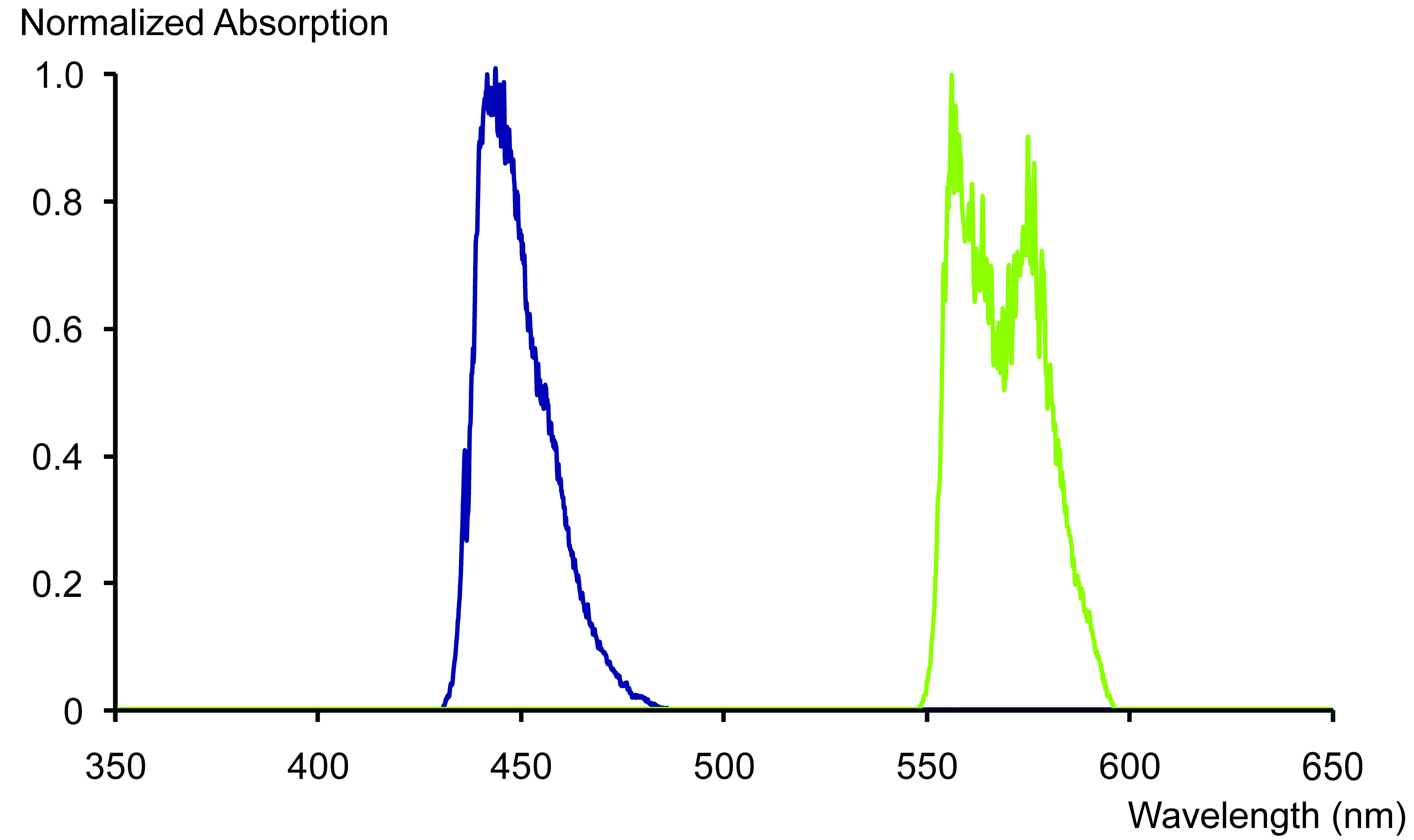

Supplement: Figure S5 — Spectra from blue and green light sources used for selective illumination. Different segments of a freely moving worm could be illuminated by sending the appropriate illumination pattern to a modified video projector that was aligned with the epi-fluorescence port of an inverted microscope (Axiovert 35, Zeiss). A 475 nm short-pass filter (UQG optics) was added in the blue path and a 568/50 nm filter (Chroma) in the green path; resulting spectra are shown. (TIF) [file pone.0040937.s005.tif]

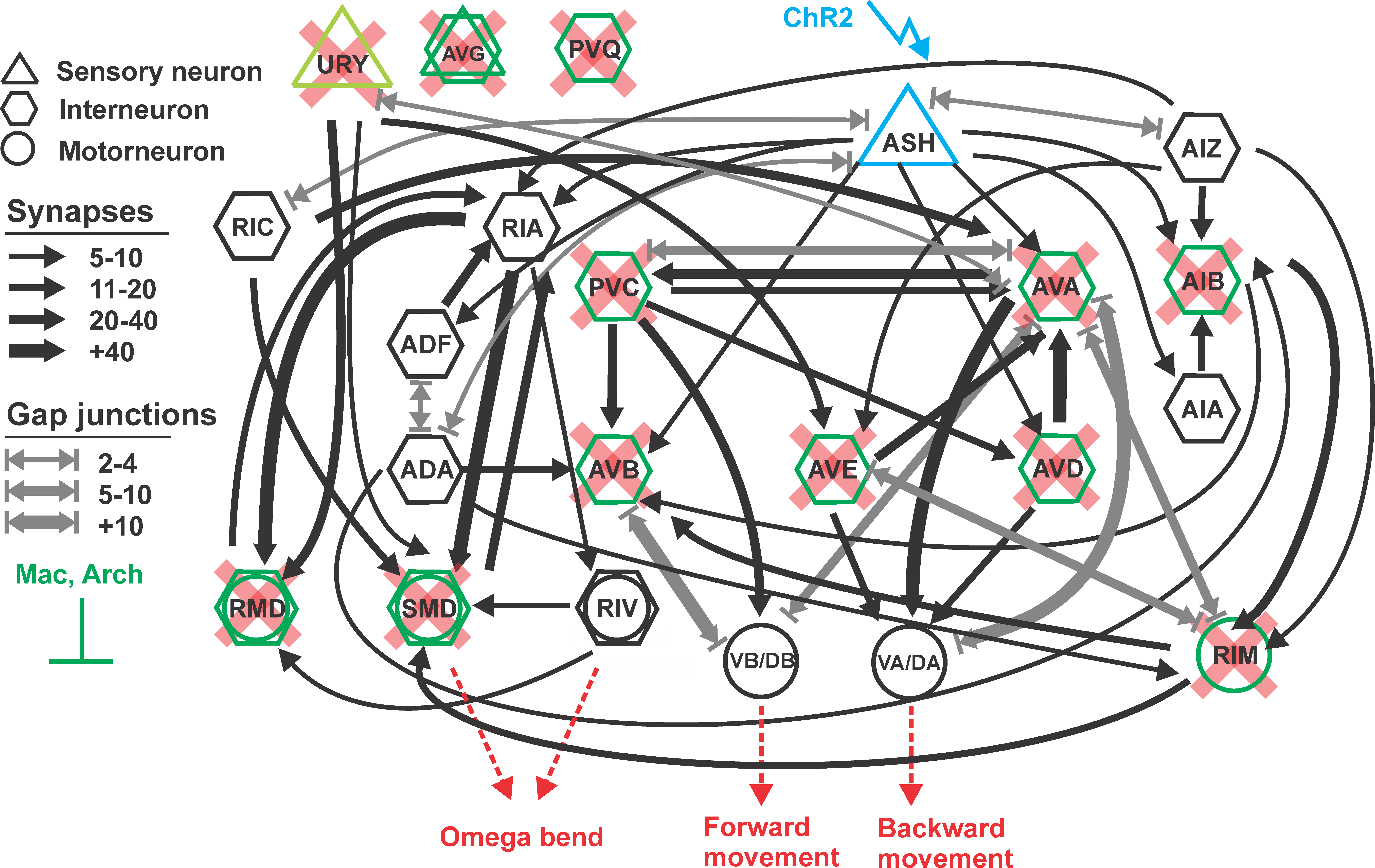

Supplement: Figure S6 — Wiring diagram of the polymodal nociceptive neuron ASH. Schematic representation of the wiring diagram of the ASH circuit with indication of the synaptic contacts and gap junctions according to [11]. (TIF) [file pone.0040937.s006.tif]
